# Facilitators and barriers to infant post-natal HIV prophylaxis, a qualitative sub-study of the PROMISE-EPI trial in Lusaka, Zambia

**DOI:** 10.3389/fpubh.2023.1242904

**Published:** 2023-08-17

**Authors:** Anaïs Mennecier, Beauty Matoka, Maria Melany Wilfred-Tonga, Catherine Chunda-Liyoka, Mwiya Mwiya, Nicolas Nagot, Jean-Pierre Molès, Philippe Van de Perre, Chipepo Kankasa, Rachel King, for the PROMISE-EPI social science sub-study group

**Affiliations:** ^1^Pathogenesis and Control of Chronic and Emerging Infections, Montpellier University, INSERM, EFS, CHU Montpellier, Montpellier, France; ^2^Pediatric Centre of Excellence, University Teaching Hospital, Lusaka, Zambia

**Keywords:** mother to child HIV transmission, qualitative analysis, post-natal prophylaxis, adherence, sub-Saharan Africa, breast-feeding

## Abstract

**Background:**

Infant post-natal prophylaxis (PNP) is used to prevent HIV transmission through breastfeeding. The WHO edited recommendations but so far there is no consensus on the duration of prophylaxis and the type of drug used depends on national guidelines. In Zambia, the national recommendations include a three-drug prophylaxis, composed of a dispersible combined tablet of zidovudine (AZT) and lamivudine (3TC) and an oral suspension of nevirapine (NVP) for 12 weeks or until the mother’s viral load is <1,000 cp/mL. The PROMISE-EPI study, modified the PNP regimen to lamivudine only, initiated at 6 weeks and continued until 12 months to all HIV exposed uninfected infants of virally unsuppressed mothers. Our aim in this analysis was to identify barriers and facilitators to this extended PNP, the keystone toward an effective prevention.

**Methods:**

Individual interviews and focus group discussion (FGD) were conducted with PROMISE-EPI participants who had received prophylaxis for their children from the national program up to 6 weeks and then lamivudine oral solution in PROMISE-EPI study. Health care providers and PROMISE-EPI staff were also interviewed. Sessions were recorded, transcribed verbatim and translated from local languages into English. An initial code-book was designed and then adapted on the basis of the emerging themes, to allow a descriptive thematic analysis.

**Results:**

More barriers to PNP adherence were identified with triple drug prophylaxis than with lamivudine. These barriers were related to the formulation and bitter taste of AZT/3TC tablets. The ready to use formulation and sweet taste of lamivudine syrup were appreciated by mothers. Extended PNP proposed in the PROMISE-EPI study was globally well accepted and strategies were found to increase adherence. Adherence to lamivudine appeared to be better than the mothers’ adherence to their own antiretroviral therapy.

**Conclusion:**

Accompanying mothers living with HIV and giving them the choice of the PNP to prevent transmission *via* breastfeeding (type of PNP regimen and extended PNP in non-adherent mothers), may be one of the keys to reducing the burden of pediatric HIV acquisition in low and middle income countries.

## 1. Introduction

Since 2012, lifelong antiretroviral therapy (ART) is internationally recommended for pregnant and breastfeeding mothers living with HIV (1). In addition to benefitting women’s health, a good adherence to ART suppresses the maternal viral load and decreases mother to child transmission (MTCT) of HIV (2). Unfortunately, ART adherence is often suboptimal, particularly during the postpartum period (3).

Post-natal prophylaxis (PNP) is internationally recommended by WHO for 6 to 12 weeks (2) to further protect HIV-exposed uninfected infants (HEU) from perinatal HIV transmission. In order to protect the infants at high-risk of HIV acquisition during breastfeeding, the 2020 Zambian national guidelines recommend the use of a three-drug prophylaxis. This PNP is initiated at birth for 12 weeks. However, in mothers with a viral load ≥1000cp/mL at 12 weeks after delivery, PNP is continued until the mother has a documented suppressed viral load or until the end of breastfeeding (4). The PNP is composed of two different formulations: (i) an oral suspension for Nevirapine (NVP) administered once daily and (ii) a combined dispersible tablet for zidovudine/lamivudine (AZT/3TC). AZT/3TC dispersible tablets have to be dissolved in 6 mL of water and depending on the child’s weight, part or all of the suspension is administered twice a day. Daily reconstitution is required to ensure the stability of the suspension.

The PROMISE-EPI randomized controlled trial was implemented in Zambia and Burkina Faso between December 2019 and October 2022. The trial tested a single-drug PNP with lamivudine which was initiated for all infants at high risk of acquisition of HIV, until the end of the breastfeeding period or the end of the study at 12 months, whichever came first. Infants were classified as high risk if maternal viral load was ≥1000cp/mL measured at the second visit of immunization (EPI-2), when the baby is 6 weeks old, or at month 6. The comparative group followed the national standard of care, described above.

There is no reliable and easy-to-implement technique to assess adherence to infant HIV PNP in resource-limited countries. Long-term PNP adherence was studied through quantitative data from survey interviews with mothers (5) or weighed returned bottles within a clinical trial (6). While these studies identify risk factors for non-adherence (5), they cannot explore reasons and context in-depth, as can be assessed in qualitative studies.

Several qualitative studies, implemented in low and middle income countries, assessed barriers to pediatric ARV adherence in children living with HIV (7–9), one focusing on infants living with HIV (9). To our knowledge, there are no similar studies on HIV PNP administered to HEU neonates and infants.

The present research is part of a larger implementation sub-study of PROMISE-EPI trial with the aim to explore the factors (1) influencing Prevention of MTCT (PMTCT) program (2) associated with the acceptability and feasibility of the standard of care compared to the PROMISE-EPI intervention. Focusing on the relationship to drugs, this analysis aimed to characterize (1) the comparison of the barriers and facilitators of the three drug prophylaxis provided by the Zambian national program versus lamivudine prophylaxis provided in PROMISE-EPI; (2) the barriers and facilitators of long term use of lamivudine.

## 2. Materials and methods

### 2.1. Context and settings

A detailed description of PROMISE-EPI intervention has been described elsewhere (10). In Zambia, the study was implemented in four health facilities of Lusaka (Chilenje, Bauleni, Matero Main and Chaisa). There were dedicated PROMISE-EPI staff members including nurses and clinical officers who cared for the participants in a dedicated study room.

Since inclusion in the PROMISE-EPI study was at 6 weeks post-partum, HEUs were assumed to be taking the three drug prophylaxis from birth as per national guidelines, until recruitment into the study. Thereafter, infants in the intervention group whose mother had an unsuppressed viral load (≥1000cp/mL) switched to lamivudine. Mothers received appropriate counseling on how to administer the drug, and monthly drug refill visits were scheduled. A syringe was provided, clearly marking the volume of drug to withdraw, according to the baby’s weight. The doses, to be given twice daily, were divided into 3 weight bands: 0.75 mL if 2 to 4 kg; 2.5 mL if 4 to 8 kg; 5 mL if >8 kg. Infants of mothers newly unsuppressed at 6 months initiated lamivudine at that particular visit. Infants in the intervention group whose mothers had a viral load <1,000 cp/mL were referred to the national program for discontinuation of the three drug prophylaxis. All children were followed until they were 12 months old.

### 2.2. Study design, participants’ sampling and selection

The study was implemented in all four PROMISE-EPI sites. In depth interviews selected in this analysis were conducted with different groups of participants: (1) PROMISE-EPI participants who experienced successively the two types of PNP, (2) Health care providers (HCP) contributing to the PMTCT program in the facilities where the study was implemented, and (3) PROMISE-EPI staff members. A purposive sampling approach was applied to select the participants in each group. Of the 87 PROMISE-EPI participants in Zambia eligible for lamivudine at either EPI-2 or 6 months, 56 could be contacted for this study because they previously consented at the inclusion to the PROMISE-EPI trial. Written informed consent were obtained from HCP and PROMISE-EPI staff members.

We conducted both individual interviews and focus group discussions (FGD) with the three types of profiles to take advantage of the strengths of both group and individual data collection methods.

Triangulation, through the involvement of the four sites, inclusion of three groups of participants and two types of data collection provided a clear picture of the barriers and facilitators of the two comparable PNP drug regimens and the extended use of lamivudine (11).

### 2.3. Data collection

PROMISE-EPI staff members, after having received training in qualitative data collection, contacted the PROMISE-EPI participants and HCP. They conducted face to face individual interviews and focus group discussions based on a semi-structured guide, lasting about 1 h each, between December 2021 and September 2022 in the PROMISE-EPI study rooms.

PROMISE-EPI participants received compensation for travel to the study site and for their time (100Kwatcha = 5 USD).

Interviews were conducted in one of the 3 main languages in Lusaka, Bemba, Nyanja or English and were audio recorded. Simultaneously translation in English was done when necessary and transcribed by the interviewer, keeping the anonymity of the interviewee by using PROMISE-EPI ID numbers (or a dedicated number for PROMISE-EPI staff and HCP).

Participants were asked about facilitators and barriers to lamivudine adherence, with respect to maternal antiretroviral (ARV) adherence and were asked to compare their experience between 3 drugs and single drug prophylaxis.

Challenges encountered by the interviewers were identified through a review of the initial transcripts by the social science researcher (RK) and discussed with the study team members. The social science researcher then participated in a sample of focus group of discussions to provide feedback on techniques. A meeting of the study team, conducted after reviewing ten transcribed interviews, determined the approximate number of additional interviews expected to reach saturation.

### 2.4. Data analysis

An initial code-book was designed using *a priori* codes from the interview and FGD guide and then adapted on the basis of the emerging themes found in five initially double-coded transcripts. These first transcripts were coded by two researchers (RK and AM) and compared to ensure reliability. Any discrepancies were discussed and resolved. The following transcripts were coded by one researcher (AM) using Excel software and reviewed by the second (RK).

A descriptive thematic analysis was chosen to explore and describe the factors leading to PNP barriers and facilitators (12). Three main themes and seven sub-themes emerged from the data and were related to the study objectives (Table 1).

**Table 1:** Themes and sub-themes.

Main themes	Sub-themes
Comparison of facilitators and barriers for two types of PNP adherence	
	Number of drugs
	Type of formulation
	Secondary effects
	Dosing device
	Novelty of the drug
Extended use of lamivudine as PNP	
	Barriers to long term adherence
	Relation to breastfeeding
Adherence to prolonged lamivudine use vs. maternal adherence to ART	

PNP: post-natal prophylaxis; ART: antiretroviral treatment.

Trustworthiness was enhanced through member checks and feedback was also obtained from dissemination with national stakeholders, the study team and PROMISE-EPI staff.

Data from PROMISE-EPI electronic Case Report Form (eCRF) were used to present the participants characteristics. Statistical analyses, including student test for mother’s age and Fisher exact test for other variables, were carried out using Stata 16.1 (Stata Corp, College Station, Texas). Missing data were omitted from percentage calculations.

### 2.5. Ethical considerations

Ethical approval for this ancillary study was obtained from Zambian IRB (ERES converge, on the 01st September 2021) and the Zambian national ethics committee (National Health Research Authority-NHRA, on the 30th September 2021).

## 3. Results

In total 17 individual interviews and eight FGD were included in this analysis. Among them, 33 PROMISE-EPI participants, eight PROMISE-EPI staff and nine HCP including nurses, counselors, lab technicians and a pharmacist participated.

The baseline characteristics of the PROMISE-EPI participants interviewed are described in Table 2. Interviewed mothers were not significantly different, in terms of basic socio-demographic characteristics, from those of lamivudine-eligible but not interviewed PROMISE-EPI participants. Nevertheless, interviewed mothers were significantly less likely to be lost to follow-up for the M12 PROMISE-EPI visit (0/32) compared to the non-interviewed mothers (15/55) (*p* < 0.001).

**Table 2:** Comparison of baseline characteristics between lamivudine-eligible PROMISE-EPI participants who were interviewed or not interviewed.

		PROMISE-EPI participants interviewed	Eligible PROMISE-EPI participants for lamivudine but not interviewed	*p* value
		*N* = 32*	*N* = 55	
Mothers				
Age	Mean (sd)	29.4 (6.9)	28.5 (5.9)	0.53
Time of HIV diagnosis	Before this last pregnancy	17/31 (53%)	26/52 (47%)	0.66
	During/after this last pregnancy	15/31 (47%)	29/52 (53%)	
Mothers not taking ART		4 (13%)	7 (7%)	0.52
ARV regimen	TDF/3TC/DTG	6/27 (22%)	10/48 (21%)	
	TDF/3TC/EFV	20/27 (74%)	38/48 (79%)	
	AZT/3TC/LPVr	1/27 (4%)	0/48 (0%)	
Highest level of education	None	2 (6%)	4 (7%)	0.22
	Primary	11 (34%)	29 (53%)	
	Secondary or more	19 (59%)	22 (40%)	
Employment situation during pregnancy	Not working	20 (63%)	40 (73%)	0.25
	Working (formal sector)	0 (0%)	3 (5%)	
	Working (informal sector)	10 (31%)	11 (20%)	
	Studying	2 (6%)	1 (2%)	
Marital status	Married/cohabitating	23 (72%)	44 (80%)	0.64
	Single	8 (25%)	10 (18%)	
	Divorced/separate/widowed	1 (3%)	1 (2%)	
HIV status known by partner	No	5/29 (17%)	7/44 (16%)	1.00
	Yes	23/29 (79%)	36/44 (82%)	
	Not applicable (no partner)	1/29 (3%)	1/44 (2%)	
Maternal viral load VL > =1000cp/ml/Infant eligible to lamivudine at EPI-2		30 (94%)	42 (76%)	0.04
Maternal viral load VL > =1000cp/ml at M6		16 (50%)	30/47 (64%)	0.25

Infants				
Sex	Female	15 (47%)	28 (51%)	0.82
Six weeks PNP at birth (3 drugs)		29 (91%)	50 (91%)	1.0
Newly eligible to lamivudine at M6 (mother newly unsuppressed)		2 (6%)	13 (24%)	0.044

* The PROMISE-EPI identifier was not recorded for one PROMISE-EPI participant. ART: antiretroviral treatment; ARV: antiretroviral; AZT: zidovudine; 3TC: lamivudine; EFV: efavirenz; LPVr: lopinavir/ritonavir; DTG: dolutegravir; TDF: tenofovir; PNP: post-natal prophylaxis.

The children and infants were between 6 and 18 months old at the time of these interviews.

### 3.1. Theme 1: comparison of facilitators and barriers to adherence for two types of PNP

**Figure 1:**
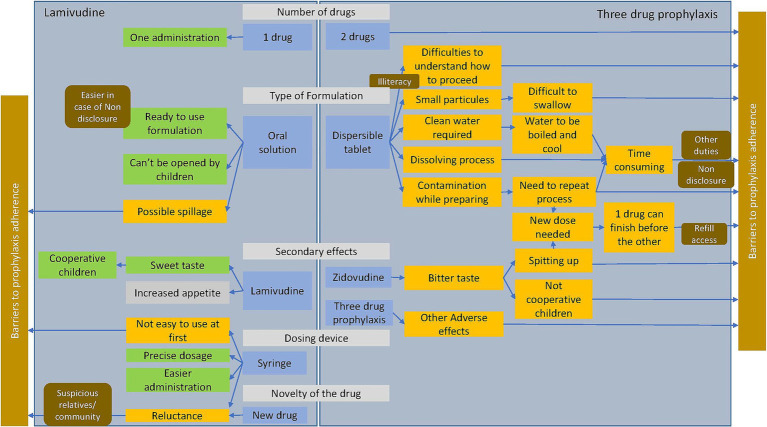
Comparison of facilitators and barriers factors of lamivudine and three drug prophylaxis adherence (in green the facilitators, in yellow the barriers, in gray the factor that can be considered either positive or negative; the context is in brown).

More barriers to PNP adherence were identified with triple drug prophylaxis than with lamivudine (Figure 1).

#### 3.1.1. Sub-theme: number of drugs

One pharmacist highlighted that, the use of a single drug facilitates adherence. Once daily (instead of twice) would be even more acceptable to mothers.

“I feel it’s a good way to go because it reduces the drug load, so it can really help mothers to improve adherence. They should even make it once daily; it can even be better.” (Pharmacist; individual interview)

#### 3.1.2. Sub-theme: type of formulation

Most mothers and HCP noted challenges with the type of AZT/3TC formulation. Interviewees unanimously expressed difficulties in measuring and preparing the oral suspension. The dosage was not always well explained by the HCP or well understood by the mothers, especially if illiterate.

“Looking at systems used in triple therapy, it’s too much to some clients, they lack that understanding to make those mixtures so, you find that adherence … is affected negatively.” (Nurse; FGD)

The drug preparation process was long. It required boiling and cooling water, when clean water was not available, and the drug to dissolve. Contamination from flies and dust was a risk during preparation, requiring the entire process to be repeated. This was a challenge for those with many other duties to be performed, and also for those who had not disclosed their HIV status.

“It used to take a lot of time for me to prepare the drug. I needed to boil water, I would wait for it to cool down, and wait for the drug to melt. I used to have a challenge, especially when I was busy with other things.” (Mother, FGD)

One mother pointed out that the tablet would not dissolve completely, leaving small particles, which make difficult for the infant to swallow.

When asked to compare, all mothers preferred the ready-to-use formulation of lamivudine. The acceptability and fidelity were notably high when there had been no disclosure to relatives. Furthermore, the oral solution is not accessible to children because they cannot open the bottle, unlike the tablets delivered as part of the triple drug prophylaxis. However, participants noted that the formulation of lamivudine could lead to spillage.

#### 3.1.3. Sub-theme: secondary-effects of both drug regimens

Both mothers and health care providers reported that the bitter AZT/3CT suspension tablet was not palatable for the children, who would often spit the medicine. Other adverse effects such as vomiting, and diarrhea were reported by several mothers with the three drug prophylaxis without specifying which drug was involved.

“The triple drug caused diarrhea and vomiting on my baby, it never used to stop. Most cases when a baby is small people will just say its normal for a baby to have diarrhea and I [also had that] thinking it was normal, but the moment the drug was changed, the diarrhea ended.” (Mother 27 yrs; FGD)

Mothers would run out of tablets of AZT/3TC tablets before nevirapine oral suspension because of having to prepare the extra dose more than once. Some mothers reported that it was challenging to refill the medication earlier than expected because of difficulties in getting to the clinic or because of bad reception by the staff. They preferred to wait until the next appointment and only give nevirapine in the meantime.

Fewer adverse events were reported with lamivudine compared to three drug prophylaxis. In addition, lamivudine oral solution’s sweet taste, increased its acceptability. Mothers observed that the lamivudine increased the baby’s appetite. It was considered an advantage by most of them because the babies gained weight and were therefore considered healthy in the community.

“Most of the times, my baby looked very healthy because of lamivudine and it made her have more appetite even if she was not feeling well, she would still eat and suck a lot.” (Mother 39 yrs; FGD)

#### 3.1.4. Sub-theme: dosing device

In PROMISE-EPI, the provision of a syringe for the lamivudine oral solution administration, although not always easy to use at first, allowed accurate dosing and facilitated administration even while the baby was sleeping, but could raise some suspicion from the community.

“You gave syringes so we cannot spill. You can even give baby while sleeping, you just put the syringe in the mouth and baby will suck it and swallow.” (Mother 30 yrs; individual interview)

#### 3.1.5. Sub-theme: novelty of the drug

Lamivudine was considered new and different from standard prophylaxis that was known in the national guidelines, which can create mistrust and rumors from relatives and community members as well as reluctance from the HCP. But as one counselor pointed out, the same reservations existed when triple drug prophylaxis was first introduced. He noted that, it is their role to educate mothers when new treatments are made available.

“Some become suspicious because of the way we give this medication using a syringe instead of maybe a spoon, and that’s why we make sure it’s just between me and my household (husband).” (Mother 25 yrs; FGD)

‘I prefer the triple drug because I have seen how it works; at least most babies have not been infected that have been on triple drug prophylaxis (Early infant diagnosis technician; individual interview)’.

### 3.2. Theme 2: extended use of lamivudine as PNP

#### 3.2.1. Sub-theme: barriers to long term adherence

Barriers to adherence reported by some mothers were: forgetfulness mainly at the time of lamivudine initiation, non-disclosure and fear of stigma.

“When it’s time to give your child medication, you will not give because of the person next to you who you have not opened up to.” (Mother 22 yrs; FGD)

One mother also reported missing doses for fear of a drug interaction when the baby was being treated for an acute illness.

The mothers, counseled by PROMISE-EPI staff, developed strategies to improve adherence to lamivudine (Table 3). To avoid forgetting a dose, they chose a specific time to give the child’s PNP that was convenient, such as, the first thing in the morning. Furthermore, giving PNP and taking ARVs at the same time ensured good adherence by the child and the mother.

**Table 3:** Barriers to extended use of lamivudine and strategies to overcome them.

Barriers	Strategies to increase adherence
Forgetfulness	Drug intake at a specific time every day for the child PNP and mothers ARVs
Non-disclosure and stigma	Help from relatives, devised tricks to hide the drug involved
Fear of interaction	Appropriate counselling

PNP: post-natal prophylaxis; ARVs: antiretrovirals.

“Some of us we do not work, we just sell by the roadside, and I make sure I do not forget to administer the medication before going for orders.” (Mother 32 yrs; FGD)

To cope with the stigma, some mothers asked for help from relatives. Others disguised the medicine, e.g., one mother confessed to filling a bottle of paracetamol with lamivudine.

“I had challenges giving the medicine at the beginning due to lack of disclosure, I got a Panadol bottle and put in lamivudine.” (Mother 39 yrs; FGD)

#### 3.2.2. Sub-theme: relation to breastfeeding

Adherence to lamivudine had an impact on breastfeeding behavior. In cases of missed doses one mother would not breastfeed for fear of endangering the child.

“For me when I forget, it means I will not breastfeed my child for fear of putting my child at risk. No matter how long my child cries, I will make sure I try by all means to help the child to stop crying without giving him the breast.” (Mother 32 yrs; FGD)

Some mothers were reassured of the protection provided by lamivudine and felt more confident to continue breastfeeding.

“What made me to breastfeed my child up to one year 1 month was, because of the medication that I was getting from the promise – EPI study for my child. If it was not for that medication, I was going to stop breastfeeding the child at 5 months.” (Mother 27 yrs; FGD)

### 3.3. Theme 3: adherence to prolonged lamivudine use vs. maternal adherence to ART

Interviewed mothers with unsuppressed viral load admitted to being more adherent to PNP for their child than to their own ARVs. Unfortunately, some mothers were not taking ARVs for their own health. For example, this mother had difficulties taking ARVs because of side effects (nausea), but had no problem giving lamivudine to her child.

“I did not have challenges when giving my child [medication], but mine, that’s where I had a challenge because I found it hard to accept.” (Mother 22 yrs; FGD)

The desire to protect their child from HIV gave them the courage to face the challenges such as the fear of stigma, or frequent visits to health facility. They would find it difficult to accept transmission through breastfeeding.

“I never wanted my child to be going through what I went through. I also did not have a challenge, whenever am going out with my baby I would administer or give medication to my baby without feeling shy.” (Mother 29 yrs; FGD)

According to PROMISE-EPI staff, some mothers suggested that, giving PNP was a more direct and easier way to protect the child from acquiring HIV than indirectly, by taking ARVs.

“For their ART it is quite a challenge, there are a lot of participants or clients who are defaulters but for their babies they are consistent because they know that it causes a risk for them not to give medicines to their babies.” (PROMISE-EPI staff; individual interview)

## 4. Discussion

Our analysis provided insight into the barriers and facilitators of extended infant PNP. The PROMISE-EPI mothers interviewed were intentionally those whose infants were at higher risk of HIV acquisition through breastfeeding due to unsuppressed maternal plasma viral load. Understanding their challenges and preferences is essential to better inform the national program and scale up promising practices. The data from the HCP and PROMISE-EPI interviews were congruent with those from the mothers, while providing a broader view.

The mothers, as well as the health care professionals, were critical of the national program’s PNP. Main barriers were due to the AZT/3TC formulation. The process of dissolving the AZT/3TC tablet has been described as time consuming, which was particularly challenging in case of non-disclosure and/or fear of stigma. In addition, the complex preparation of AZT/3TC tablets may have contributed to inadequate dosing. Manipulation and calculation errors may occur with pediatric formulations, particularly with small volume administration and pre-dilution (13). WHO insists on minimizing manipulation before administration and warns about the risk associated with incorrect dosing, especially when a precise dose is required (14). Because of this dosing challenge, the oral liquid formulation of ART is internationally recommended for newborns living with HIV, and to switch as soon as possible to a solid oral dosage, such as dispersible tablets (2). Better stability, uniform dosage, options for different doses, convenient packaging and ease of transport are the advantages highlighted by WHO over the liquid oral formulation (14).

The AZT/3TC bitter taste, sometimes associated with regurgitation had a negative impact on child cooperation. Poor palatability had already been largely reported as an obstacle to successful administration of pediatric drugs (15–17) and this aspect is considered by EMA (European Medicines Agency) and WHO as one of the main elements to evaluate the patient acceptability (14, 18).

We found that time consuming, complex preparation and poor palatability of the AZT/3TC tablets had a negative impact on the drug acceptability, which likely had resulted in poor adherence. In comparison, the single drug and the ready-to-use lamivudine oral solution were considered to facilitate good adherence. The provision of a syringe, accompanied by instructions and practice of how to draw the medicine, was appreciated by the mothers. This facilitated the ease of administration of the drug, even when the child was sleeping. In addition, the availability of an appropriate dosing device reduces the risk of incorrect dosing (14).

In a study of infants living with HIV where ARVs were given in syrup and then in tablets when they reached 12 kg, tablets were preferred to syrup by caregivers and children themselves. The main reason for this preference was related to the practical issues of syrup, such as transportation and conspicuousness (19). Acceptability is related to context including age of infants/children and treatment duration. In our context, the dispersible tablets (combined with an oral suspension) were initiated at birth, which may have created additional difficulties compared with taking them in children. On the other hand, taking ART for life for children living with HIV does not have the same implications as taking HIV PNP for a limited period of time, even if it is extended to the entire breastfeeding period.

Prolonged lamivudine has already proved effective and safe in previous studies (6, 20, 21), and is considered a currently available option for PNP by the WHO (22), although not recommended in its guidelines. Its extended use in PROMISE-EPI was generally well accepted by the participants interviewed. The good adherence would have contributed to the sharp reduction in HIV transmission in the trial intervention arm compared to the comparative arm (23). Identified barriers to the extended use of lamivudine PNP in our analysis, such as non-disclosure, fear of stigma, or forgetfulness, are among those known to affect maternal adherence to ARVs (24–27). Evidence of strategies implemented to overcome these barriers demonstrate the importance of understanding and providing mothers with appropriate counseling to ensure a good PNP adherence. These same approaches have been shown to increase maternal adherence to ARVs (28, 29). Interestingly, adherence to long term lamivudine had an impact on the duration of breastfeeding for some mothers, as well as on their adherence to ART. This argues in favor of continuing PNP until the end of breastfeeding in children whose mothers are unsuppressed at some point, even if they become suppressed later, as being done in the PROMISE-EPI study, but not in the Zambian guidelines (4).

ARV non-adherence can be considered as the main reason for unsuppressed viral load according to previous studies (30, 31). While interviewed mothers admitted to not being adherent to their ARVs, they reported being generally adherent for their child’s PNP within PROMISE-EPI study. The direct action through the administration of lamivudine seemed to make them more responsible than the indirect action through their ARV intake for their own health. Although extended use of PNP is not internationally recommended, several national guidelines among the priority countries for eliminating MTCT have opted for this method to protect infants from HIV transmission throughout the breastfeeding period (32, 33). Given the difficulty of overcoming viral suppression in some breastfeeding mothers, in particular when viral load monitoring is not optimal, as is the case in low and middle income countries, extended PNP can be the key to avoiding HIV transmission. Nevertheless, the choice of a multiple drug extended prophylaxis, as recommended in several countries with high HIV burden (32, 33), is questionable. Indeed, the combination of drugs complicates the regimen, which may be the cause of poor adherence, especially in mothers who have difficulties in adhering to their own HIV treatment. Poor adherence to PNP in high-risk infants is problematic because exposed them to drug resistance mutation in the event of breakthrough infection (34).

Limitations were identified in our study, including one related to the conduct of the interviews by the PROMISE-EPI staff. As they followed the participants for several months within the parent study, there was mutual trust, it was easier for staff to reach out to the mothers and for the mothers to express themselves freely. However, this may have induced a social desirability bias. Moreover, those who agreed to be interviewed were more likely to have appreciated the intervention proposed in PROMISE-EPI. This is confirmed by the significant difference in the rate of lost to follow up for the PROMISE-EPI M12 visit between interviewed and non-interviewed mothers.

Providing mothers living with HIV with appropriate counseling and giving them the choice of tools to prevent HIV transmission *via* breastfeeding (type of PNP regimen and extended PNP in non-adherent mothers) may be one of the keys to reducing the burden of pediatric transmission in low-and middle-income countries.

## Data availability statement

The raw data supporting the conclusions of this article will be made available by the authors, without undue reservation.

## Ethics statement

The studies involving humans were approved by ERES converge and Zambian National Health Research Authority. The studies were conducted in accordance with the local legislation and institutional requirements. Written informed consent for participation in this study was provided by the participants’ legal guardians/next of kin.

## PROMISE-EPI social science sub-study group

Pathogenesis and Control of Chronic and Emerging Infections, Montpellier University, INSERM, EFS; CHU Montpellier, (France): RK (principal co-investigator of the PROMISE-EPI social science sub-study); PV (principal investigator of PROMISE-EPI); NN (methodologist); J-PM (international laboratory coordinator); AM (international project manager); Morgana d’Ottavi (central data-manager and biostatistician).

Pediatrics Centre of Excellence, University Teaching Hospital, Lusaka (Zambia): CK (principal co-investigator for PROMISE-EPI and the social-science sub-study); MM (project coordinator); CC-L (assistant coordinator); MW-T (medical officer social science sub-study management); David Rutagwera (laboratory coordinator); BM (monitor and social science sub-study management); Sylvester Banda (study nurse, social science sub-study organization and interviewer); Faith Sitali (site investigator and interviewer); Chayson Maunda (site investigator and interviewer); Mwape Kelvin Chisala (site investigator and interviewer); Richard Kandela (site investigator and interviewer); Kennedy Changwa Sikambale (study nurse and interviewer); Mwape Chibale (study nurse and interviewer); Sara Phiri (study nurse and interviewer); Gertrude Munanjalaa (study nurse and interviewer); Vera Ndulumina Kawanga (study nurse and interviewer); Eric Maseko Phiri (study nurse and interviewer); Shanzi Mulenga (study nurse and interviewer); Jenala Nyirenda Hapenga (study nurse and interviewer); Kapambwe Mulenga (study nurse and interviewer).

## Author contributions

CK, RK, and AM: study conception, planning, and design. BM, MW-T, CC-L, and AM: study management. BM and MW-T: field coordination. AM and RK: data coding and analysis. AM: drafting of the manuscript. CK, CC-L, RK, PV, and J-PM: editing the manuscript. CK, CC-L, MM, MW-T, BM, PV, NN, AM, and J-PM: main contributors of PROMISE-EPI study. All authors significantly contributed to the manuscript and approved the final version of the manuscript.

## Funding

This study was funded by Ministère de l’Europe, et des affaires étrangères (France) (French Ministry of Foreign Affairs) and Sidaction: grant number: 21-1-AEQ-12981.

## Conflict of interest

The authors declare that the research was conducted in the absence of any commercial or financial relationships that could be construed as a potential conflict of interest.

## Publisher’s note

All claims expressed in this article are solely those of the authors and do not necessarily represent those of their affiliated organizations, or those of the publisher, the editors and the reviewers. Any product that may be evaluated in this article, or claim that may be made by its manufacturer, is not guaranteed or endorsed by the publisher.
